# Outcomes of Radiocephalic Arteriovenous Fistulas (RC-AVFs) in Patients Aged ≥70 Years: The Impact of Frailty on Maturation and Patency

**DOI:** 10.7759/cureus.111124

**Published:** 2026-06-18

**Authors:** Alaa Hassouneh, Rajinder Singh, Laveena Castelino, Babatunde Campbell

**Affiliations:** 1 Vascular Surgery, Manchester University NHS Foundation Trust, Manchester, GBR; 2 Transplant Surgery, Manchester University NHS Foundation Trust, Manchester, GBR

**Keywords:** arteriovenous access for renal dialysis, arteriovenous fistula, distal radiocephalic fistula, elderly population, radiocephalic fistula, transplant dialysis

## Abstract

Background

Radiocephalic arteriovenous fistulas (RC-AVFs) are preferred for hemodialysis access, but outcomes in elderly patients remain debated. Frailty may influence maturation and patency.

Methods

This was a retrospective cohort study of patients aged ≥70 years who underwent RC-AVF creation over a 12-month period at a single center. Frailty was assessed using the Clinical Frailty Scale (CFS) and categorized as frail (CFS ≥5) or non-frail (CFS ≤4). The primary outcome was primary failure (failure to achieve functional hemodialysis use during follow-up). Secondary outcomes included patency at three, six, and 12 months and the need for intervention. Logistic regression was used to assess the association between CFS (per one-point increase) and primary failure.

Results

A total of 52 patients were included (mean age: 75.9 years; 38 {73.1%} male). Primary failure occurred in 23 (44.2%) patients. Frail patients had higher rates of primary failure than non-frail patients (12/20 {60.0%} vs. 11/32 {34.4%}, p=0.090). Each one-point increase in CFS was associated with increased odds of primary failure (OR: 1.87, p=0.013). Patency at three, six, and 12 months was 44 (84.6%), 39 (75.0%), and 29 (55.8%), respectively; 12-month patency was lower in frail patients (4/11 {36.4%} vs. 12/18 {66.7%}, p=0.143). Interventions were required in 17 (32.7%) patients, most commonly angioplasty.

Conclusions

Frailty was strongly associated with primary failure after RC-AVF creation in patients aged ≥70 years. Incorporating frailty assessment into preoperative evaluation may improve patient selection and counseling, and support shared decision-making.

## Introduction

Radiocephalic arteriovenous fistulas (RC-AVFs) are considered the preferred form of vascular access for hemodialysis due to their superior long-term patency and lower complication rates compared with alternative access options. However, outcomes in elderly patients remain debated, particularly due to concerns regarding higher rates of non-maturation and early failure [1]. As the global population ages, the number of older patients with end-stage renal disease (ESRD) requiring renal replacement therapy continues to rise, making the establishment of reliable and durable vascular access increasingly important [2].

Older patients often present with comorbidities, such as diabetes, vascular disease, and reduced vessel quality, all of which may negatively impact AVF maturation and longevity [3]. In this context, careful patient selection and individualized access planning are essential. Contemporary vascular access guidance increasingly supports a patient-centered approach, such as the end-stage kidney disease (ESKD) Life-Plan, in which access decisions are tailored to patient characteristics, life expectancy, comorbidities, anatomy, and goals of care [4]. Despite this, chronological age alone is a poor predictor of outcomes, and there is growing interest in more comprehensive measures of physiological reserve [5].

Frailty, defined as a state of reduced physiological reserve and increased vulnerability to stressors, has emerged as an important predictor of adverse outcomes across a range of surgical and medical settings [6]. The pathophysiological processes underlying frailty, including inflammation, sarcopenia, and impaired vascular function, are highly prevalent in patients with ESRD, and frailty is common among individuals receiving hemodialysis [7,8]. Emerging evidence suggests that frailty may influence vascular access outcomes, including AVF maturation, patency, and the need for reintervention [9,10]. Recent work has also shown that frailty is associated with increased reintervention after AVF creation, supporting its potential role in vascular access risk stratification [11].

## Materials and methods

Study design and population

This retrospective cohort study included consecutive patients aged ≥70 years who underwent primary radiocephalic arteriovenous fistula (RC-AVF) (end-to-side anastomosis with distal venous ligation) creation for hemodialysis access over a 12-month period at a single tertiary vascular center. Patients undergoing revision procedures or alternative access (e.g., brachiocephalic or grafts) were excluded. The study was conducted in accordance with institutional guidelines; ethical approval was waived due to the retrospective design and use of anonymized data.

Data collection

Demographic, clinical, and operative data were extracted from electronic patient records. Variables collected included age, sex, body mass index (BMI), comorbidities (including diabetes mellitus and cardiovascular disease), and relevant medication history where available. Preoperative vascular assessment findings, including vessel diameter measurements from duplex ultrasound (where performed), were recorded. Operative details included anesthetic technique (local vs. regional block) and laterality.

Frailty assessment

Frailty was assessed using the Clinical Frailty Scale (CFS), a nine-point, clinical judgment-based frailty measure originally described by Rockwood et al. and validated for use in clinical practice [12]. Patients were categorized as frail (CFS ≥5) or non-frail (CFS ≤4). In addition, CFS was analyzed as a continuous variable to assess its association with outcomes.

Outcomes

The primary outcome was primary failure, defined as the RC-AVF failing to achieve functional use for hemodialysis during the follow-up period. Secondary outcomes included AVF patency at three, six, and 12 months following creation, and the requirement for any intervention to maintain or restore access function. Patency was assessed using ultrasound, supported by follow-up documentation and dialysis access records where available. Patency analyses were performed on patients with available follow-up data at each time point.

Statistical analysis

Continuous variables were assessed for normality and presented as mean±standard deviation or median (interquartile range), as appropriate. Categorical variables were presented as frequencies and percentages. Comparisons between frail and non-frail groups were performed using the chi-square test or Fisher’s exact test for categorical variables, and the independent samples t-test or Mann-Whitney U test for continuous variables, as appropriate. Logistic regression analysis was performed to evaluate the association between frailty (CFS, per one-point increase) and primary failure. Results were reported as odds ratios (ORs) with corresponding p-values. A two-sided p<0.05 was considered statistically significant. Statistical analyses were performed using standard statistical software.

## Results

**Figure 1:**
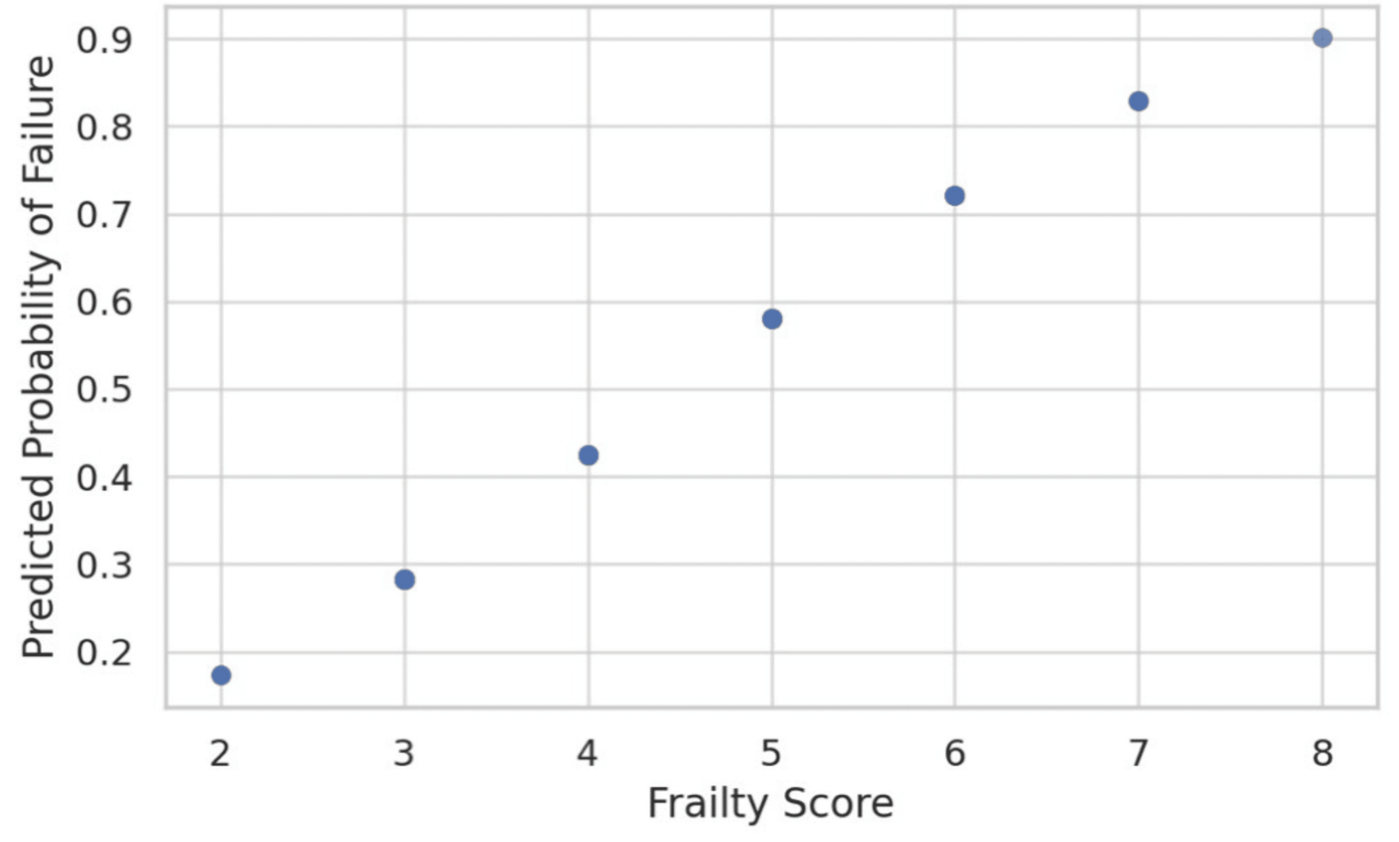
Predicted probability of primary failure by frailty score. Predicted probability of primary failure according to Clinical Frailty Scale (CFS) score based on logistic regression (per one-point increase).

**Figure 2:**
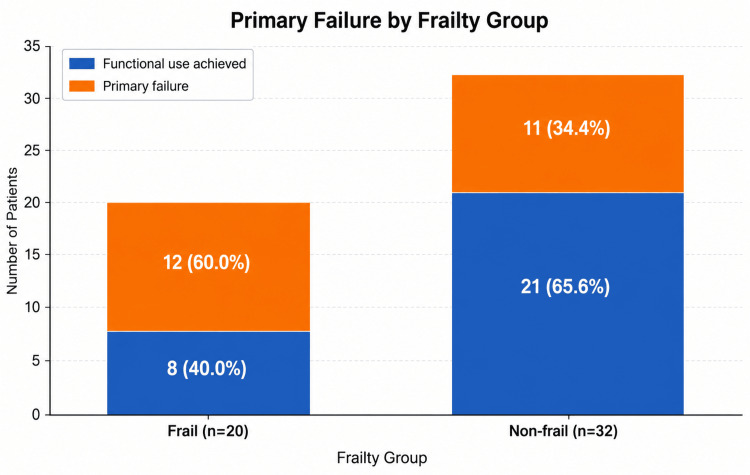
Primary failure by frailty group. Stacked bar chart showing the number and percentage of patients with and without primary failure following RC-AVF creation, stratified by frailty status (CFS ≥5 vs. CFS ≤4). RC-AVF: radiocephalic arteriovenous fistula; CFS: Clinical Frailty Scale

Fifty-two patients were included (mean age: 75.9 years, range: 70-89 years) in this study, of whom 38 (73.1%) were male. The mean BMI was 28.6, and 23 (44.2%) patients had diabetes (Table 1). Primary failure occurred in 23 (44.2%) cases. Frail patients had a higher rate of primary failure than non-frail patients, although this did not reach statistical significance: 12/20, 60.0% vs. 11/32, 34.4%; OR 2.86, 95% CI: 0.90-9.08; Fisher's exact p=0.090. Univariable logistic regression demonstrated that each one-point increase in CFS was associated with higher odds of primary failure (unadjusted OR: 1.87 per 1-point increase, p=0.013) (Figures 1, 2).

**Table 1:** Baseline demographics and comorbidities. Values are presented as n (%). This table describes baseline characteristics of the overall cohort. TIA: transient ischemic attack; CFS: Clinical Frailty Scale

Variables	n (%)
Male	38 (73)
Female	14 (27)
Frail (CFS ≥5)	20 (38)
Non-frail (CFS ≤4)	32 (62)
Diabetes	23 (44)
Hypertension	41 (79)
Ischemic heart disease (IHD)	15 (29)
Peripheral vascular disease (PVD)	8 (15)
Stroke or TIA	5 (10)

Patency at three, six, and 12 months was 44 (84.6%), 39 (75.0%), and 29 (55.8%), respectively. A total of 12-month patency was lower in frail patients than in non-frail patients, although this did not reach statistical significance as follows: 4/11, 36.4% vs. 12/18, 66.7%; OR: 0.29, 95% CI: 0.06-1.38; Fisher’s exact p=0.143 (Table 2).

**Table 2:** Factors associated with primary failure and 12-month patency. *Statistically significant results. Categorical variables were compared using Fisher’s exact test because of the small sample size. Odds ratios are reported with 95% confidence intervals. Statistical significance was defined as p<0.05. In this table, diabetes and 12-month patency were the only statistically significant associations (p=0.0018).

Outcome	Group	Events/total	%	Statistical test	OR	95% CI	p-Value
Primary failure	Frail	12/20	60	Fisher’s exact test	2.86	0.90-9.08	0.09
	Non-frail	11/32	34.4				
12-month patency	Frail	4/11	36.4	Fisher’s exact test	0.29	0.06-1.38	0.143
	Non-frail	12/18	66.7				
12-month patency	Diabetic	7/23	30.4	Fisher’s exact test	0.14	0.04-0.48	0.0018*
	Non-diabetic	22/29	75.9				
Primary failure	Diabetic	12/23	52.2	Fisher’s exact test	1.79	0.59-5.42	0.402
	Non-diabetic	11/29	37.9				

Diabetes was significantly associated with reduced 12-month patency as follows: 7/23 (30.4%) in diabetic patients vs. 22/29 (75.9%) in non-diabetic patients; Fisher’s exact p=0.0018. Diabetes was also associated with a numerically higher primary failure rate, although this was not statistically significant as follows: 12/23 (52.2%) vs. 11/29 (37.9%); Fisher’s exact p=0.402. Anesthetic technique (local vs. block) did not significantly affect patency outcomes. Interventions were required in 17 (32.7%) patients, most commonly angioplasty.

## Discussion

In this cohort of patients aged ≥70 years undergoing RC-AVF creation, primary failure was common (23/52, 44.2%), despite acceptable short-term patency (44/52, 84.6% at three months and 39/52, 75.0% at six months), declining to 29/52 (55.8%) at 12 months. These findings suggest that RC-AVF remains feasible in selected older patients; however, the risk of non-maturation or early failure is substantial and should inform counseling and access planning. This is consistent with previous literature showing that the key issue in older patients is not simply whether an RC-AVF can be surgically created, but whether it matures and becomes functionally usable for hemodialysis; this is influenced by age, comorbidity, vascular anatomy, and competing risks [13].

In this cohort, frail patients had a numerically higher rate of primary failure than non-frail patients as follows: 12/20 (60.0%) vs. 11/32 (34.4%), although this categorical comparison did not reach statistical significance (Fisher’s exact p=0.090). However, when frailty was analyzed as a continuous variable, each one-point increase in CFS was associated with higher odds of primary failure on univariable logistic regression (unadjusted OR: 1.87 per one-point increase, p=0.013). These findings suggest that increasing frailty burden may be associated with poorer RC-AVF outcomes, although the results should be interpreted cautiously given the small sample size and lack of multivariable adjustment. Frailty has increasingly been recognized as an important predictor of adverse vascular access outcomes, including higher rates of reintervention after AVF creation and potentially reduced functional access use [11].

Although RC-AVFs are conventionally preferred, their role in older patients remains contentious. A UK Cambridge series of patients aged >70 years supported an RC-first strategy, reporting acceptable one-year outcomes, with primary patency approximately 54%, secondary patency approximately 66%, and a lower incidence of steal syndrome compared with brachiocephalic fistulas [1]. In our cohort, 12-month patency was comparable at 55.8%, but primary failure remained high at 44.2%, highlighting the need for improved risk stratification within this age group. Notably, the Cambridge series also acknowledged that clinical decision-making already considers perceived frailty and life expectancy; our findings build on this by demonstrating an association between CFS and primary failure [1].

In contrast, other large-cohort data have reported less favorable outcomes for distal configurations in older patients. In an age-stratified study comparing patients aged <65 years, 65-79 years, and >80 years, older patients had inferior patency overall, and distal radiocephalic fistulas demonstrated higher non-maturation and poorer patency than more proximal configurations; distal access was also associated with loss of secondary patency [14]. These contrasting findings suggest that chronological age alone does not reliably predict RC-AVF success and that outcomes are influenced by differences in patient factors, anatomy, and competing risks.

This uncertainty aligns with the shift toward individualized vascular access planning, including the end-stage kidney disease (ESKD) Life-Plan approach, in which patient characteristics and goals guide access choice rather than a one-size-fits-all hierarchy [4,15]. In this context, frailty may serve as a clinically practical marker of biological reserve to inform decision-making. Recent evidence suggests that frailty screening can influence access planning, and frailer patients experience a higher burden of reintervention after AVF creation [16,11]. Our findings are consistent with this literature and suggest that frailty assessment may help identify which older patients are most and least likely to benefit from an RC-first approach.

Incorporating frailty assessment into preoperative evaluation could be achieved with minimal additional burden. Recording the Clinical Frailty Scale at the time of access planning, alongside ultrasound vessel mapping and dialysis trajectory, may support risk stratification and shared decision-making. Rather than acting as an absolute contraindication, a higher CFS could prompt enhanced counseling regarding the likelihood of non-maturation and potential need for reintervention, consideration of alternative autogenous configurations where appropriate, and closer early surveillance with a low threshold for imaging and salvage interventions. This approach is consistent with contemporary guidance that vascular access planning should be individualized and should account for likely complications, future access options, and patient goals [4,15]. CFS should therefore be regarded as an additional risk-stratification tool and should not be interpreted in isolation; vascular anatomy, comorbidities, nutritional status, BMI, cardiac function, dialysis trajectory, and patient goals should also be considered when planning vascular access.

This study has limitations that should be considered when interpreting the findings. First, it was a retrospective single-center study with a relatively small sample size, which may limit generalizability and reduce statistical power for subgroup analyses. Second, although CFS was recorded preoperatively as part of routine anesthetic assessment, the retrospective design means the study depended on the completeness of clinical documentation. Third, several potentially important confounders were either incompletely available or not included in the present analysis, including smoking status, markers of malnutrition, radial artery and cephalic vein diameter, and cardiac function such as left ventricular ejection fraction. These factors may contribute to frailty and may also independently affect RC-AVF maturation, patency, primary failure, and the need for reintervention. Therefore, although this study focused specifically on frailty as a clinically practical risk-stratification measure, future prospective studies with larger cohorts should incorporate anatomical, nutritional, cardiovascular, and lifestyle-related variables to better define frailty's independent contribution to RC-AVF outcomes.

## Conclusions

In this retrospective cohort of patients aged ≥70 years undergoing RC-AVF creation, increasing CFS was associated with higher odds of primary failure on univariable analysis. Although frail patients had numerically higher primary failure and lower 12-month patency than non-frail patients, these categorical comparisons did not reach statistical significance. These findings suggest that CFS may provide additional information during preoperative risk assessment, but it should not be interpreted in isolation. Frailty assessment should be considered alongside vascular anatomy, comorbidities, dialysis trajectory, and patient goals when planning vascular access. Larger prospective studies are required to validate these findings and define the independent role of frailty in RC-AVF outcomes.

## References

[1] Goh MA, Ali JM, Iype S, Pettigrew GJ (2016). Outcomes of primary arteriovenous fistulas in patients older than 70 years. J Vasc Surg.

[2] Bessias N, Paraskevas KI, Tziviskou E, Andrikopoulos V (2008). Vascular access in elderly patients with end-stage renal disease. Int Urol Nephrol.

[3] Wan ZM, Hu B, Lai QQ, Gao XJ, Tu B, Zhou Y, Zhao WB (2020). Radial artery diameter and age related functional maturation of the radio-cephalic arteriovenous fistula [sic]. BMC Nephrol.

[4] Lok CE, Huber TS, Lee T (2020). KDOQI clinical practice guideline for vascular access: 2019 update. Am J Kidney Dis.

[5] Liu S, Wang Y, He X, Li X (2024). Construction and evaluation of a predictive nomogram for identifying premature failure of arteriovenous fistulas in elderly diabetic patients. Diabetes Metab Syndr Obes.

[6] Fried LP, Ferrucci L, Darer J, Williamson JD, Anderson G (2004). Untangling the concepts of disability, frailty, and comorbidity: implications for improved targeting and care. J Gerontol A Biol Sci Med Sci.

[7] Wu PY, Chao CT, Chan DC, Huang JW, Hung KY (2019). Contributors, risk associates, and complications of frailty in patients with chronic kidney disease: a scoping review. Ther Adv Chronic Dis.

[8] Bao Y, Dalrymple L, Chertow GM, Kaysen GA, Johansen KL (2012). Frailty, dialysis initiation, and mortality in end-stage renal disease. Arch Intern Med.

[9] Makary MA, Segev DL, Pronovost PJ (2010). Frailty as a predictor of surgical outcomes in older patients. J Am Coll Surg.

[10] McAdams-DeMarco MA, Law A, Salter ML (2013). Frailty as a novel predictor of mortality and hospitalization in individuals of all ages undergoing hemodialysis. J Am Geriatr Soc.

[11] Stavert B, Monaro S, Naganathan V, Aitken S (2023). Frailty predicts increased risk of reintervention in the 2 years after arteriovenous fistula creation. J Vasc Access.

[12] Rockwood K, Song X, MacKnight C, Bergman H, Hogan DB, McDowell I, Mitnitski A (2005). A global clinical measure of fitness and frailty in elderly people. Can Med Assoc J.

[13] Lomonte C, Forneris G, Gallieni M (2016). The vascular access in the elderly: a position statement of the Vascular Access Working Group of the Italian Society of Nephrology. J Nephrol.

[14] Misskey J, Faulds J, Sidhu R, Baxter K, Gagnon J, Hsiang Y (2018). An age-based comparison of fistula location, patency, and maturation for elderly renal failure patients. J Vasc Surg.

[15] Lok CE, Rajan DK (2022). KDOQI 2019 vascular access guidelines: what is new. Semin Intervent Radiol.

[16] McDonnell SM, Nikfar S, Blecha M, Halandras PM (2024). Frailty screening for determination of hemodialysis access placement. J Vasc Surg.

